# Enhancing nurses’ competence in mechanical ventilation: a quasi-experimental study and its association with patient severity using APACHE II

**DOI:** 10.3389/fmed.2026.1891958

**Published:** 2026-08-27

**Authors:** Noha Mohammed Ibrahim, Samia Eaid Elgazzar, Amany Ibrahim Ezz Eldin, Abdo Mansoor Alhusami, Mona Metwally El-Sayed, Mohamed Goda Elbqry, Fatma Mohamed Elmansy, Shereen Ahmed A. Qalawa, Nasiru Mohammed Abdullahi, Saddam Ahmed Al-Ahdal, Hanadi Dakhilallah, Sheren Ebrahim El-Hag El-Tahry

**Affiliations:** 1 Department of Nursing, College of Applied Medical Sciences, University of Bisha, Bisha, Saudi Arabia; 2 Department of Medical-Surgical Nursing, Faculty of Nursing, Port-Said University, Port Said, Egypt; 3 Department of Medical-Surgical Nursing, College of Nursing, Qassim University, Buraydah, Saudi Arabia; 4 Department of Medical-Surgical Nursing, Inaya College of Nursing, Inaya Medical Colleges, Riyadh, Saudi Arabia; 5 Department of Nursing, College of Medicine and Health Science, Thamar University, Dhamar, Yemen; 6 Department of Community, Psychiatric, and Mental Health Nursing, College of Nursing, Qassim University, Buraydah, Saudi Arabia; 7 Department of Nursing, Medical City, Qassim University, Buraydah, Saudi Arabia; 8 Department of Medical-Surgical Nursing, Faculty of Nursing, Suez Canal University, Ismailia, Egypt; 9 Department of Nursing Administration and Education, College of Nursing, Imam Mohammad Ibn Saud Islamic University (IMSIU), Riyadh, Saudi Arabia; 10 Department of Medical Surgical Nursing, Faculty of Nursing, Mansoura National University, Mansoura, Egypt

**Keywords:** APACHE II score, competence, critical care nursing, mechanical ventilation, nursing education

## Abstract

**Background:**

Mechanical ventilation is a cornerstone of intensive care, requiring ICU nurses to have adequate knowledge, competence, and skills to provide safe, evidence-based care. Although educational programs improve nurses’ preparedness, evidence of their impact on patient outcomes remains limited.

**Aim:**

To assess ICU nurses’ knowledge, professional competence, and clinical practice related to mechanical ventilation following participation in a structured educational program, and to explore the association between nursing competence and patient severity as measured by APACHE II scores.

**Methods:**

A quasi-experimental study design was conducted in the Egyptian Health Care Authority Hospitals in Port Said Governorate. A convenience sample was used. The study involved one group of ICU nurses evaluated pre- and post-educational program, and two separate cohorts of mechanically ventilated patients (50 each) admitted before and after intervention. Nurses and patients were not paired, and patient cohorts differed in case-mix profiles. Four validated assessment tools were utilized to measure nurses’ knowledge, competence, clinical skills & patients’ demographic and clinical data.

**Results:**

ICU nurses demonstrated significantly higher post-training scores in knowledge, professional competence, and clinical performance compared with their pre-training scores (*p* < 0.001). Moreover, a substantial contrast in APACHE II scores was noted between patient groups before and after the intervention (*p* < 0.001). Nevertheless, given that APACHE II evaluates the severity of illness upon ICU admission within the initial 24 h and the patient cohorts were distinct, the observed difference is more likely due to diversities in case complexity rather than an impact directly linked to the educational program.

**Conclusion:**

Participation in the educational program was associated with higher immediate post-training scores in ICU nurses’ knowledge, professional competence, and clinical performance. The APACHE II scores of the pre- and post-intervention patient cohorts differed; however, this does not prove that the educational program had a direct impact on patient outcomes. Further multicenter studies with controlled designs are needed to better evaluate the relationship between educational interventions and patient outcomes.

## Introduction

Mechanical ventilation (MV) stands as a prevalent life-sustaining measure in intensive care units (ICUs) for individuals facing acute respiratory failure and various critical conditions. Despite its life-preserving nature, MV is linked to severe ramifications such as ventilator-associated pneumonia, ventilator-induced lung damage, extended ICU residency, and escalated fatality rates. Among the diverse ICU team, critical care nurses hold a pivotal position in conducting ongoing bedside supervision, promptly identifying complications, monitoring ventilator functionality, executing evidence-based practices, and liaising with medical practitioners and respiratory therapists to enhance patient outcomes (1–4).

Proficient management of mechanically ventilated individuals necessitates nurses to demonstrate proficient knowledge, critical clinical decision-making abilities, and adept practical expertise. Competencies in these areas are crucial for overseeing ventilator performance, recognizing patient-ventilator discordance, administering sedatives, facilitating weaning, and averting ventilation-associated issues. However, research findings have consistently underscored disparities between nurses’ theoretical acumen and hands-on application in this domain, underscoring the necessity for structured educational programs to enhance proficiency in the management of mechanical ventilation (5–13).

Numerous educational interventions have shown enhanced knowledge and clinical skills among nurses in the context of mechanical ventilation. The implementation of structured educational initiatives and training based on protocols has proven beneficial in enhancing the quality of ventilator-related care procedures. Nonetheless, prior research has predominantly focused on assessing educational achievements of nurses, with scant exploration on whether advancements in nursing proficiency correlate with quantifiable measures of patient acuity. Consequently, a critical gap exists in the literature concerning the link between improved nursing competence and standardized assessments of patient severity in ICU settings involving mechanical ventilation (14, 15).

The Acute Physiology and Chronic Health Evaluation II (APACHE II) score serves as a validated prognostic instrument extensively applied for evaluating the severity of illnesses and forecasting mortality rates in critically ill individuals within the initial 24 h of their admission to the Intensive Care Unit (ICU). Despite its primary focus on the physiological condition of patients rather than nursing efficacy, when viewed in conjunction with nursing-related metrics, the APACHE II score can offer supplementary insights into patient acuity. Research examining potential correlations between enhancements in nursing proficiency and alterations in indicators of patient severity, while considering the impact of various clinical and medical variables on APACHE II scores, remains limited (16, 17).

Therefore, the goal of this quasi-experimental study was to assess the knowledge, professional competence, and clinical practice of critical care nurses related to mechanical ventilation following participation in a structured educational program. The study also examined the APACHE II scores of the separate patient cohorts before and after the intervention. Differences in APACHE II scores were analyzed descriptively and were not regarded as proof of a direct impact of the educational program on patient outcomes because illness severity is influenced by a variety of patient- and treatment-related factors beyond nursing care alone.

## Theoretical framework

The current investigation was primarily guided by the Donabedian Quality Framework, a well-established paradigm for assessing healthcare quality that comprises three interconnected elements: structure, process, and outcomes. In this model, the educational intervention served as the process component, the proficiency of nurses in managing mechanical ventilation represented the quality-of-care provision, and patient outcomes were assessed utilizing standardized metrics such as APACHE II scores (18–23).

The optimal management of mechanical ventilation necessitates ongoing evaluation, evidence-based decision-making, and effective interdisciplinary collaboration involving nurses, physicians, and respiratory therapists within the intensive care unit (ICU) team. Higher levels of knowledge, professional competence, and clinical performance may be linked to structured training programs that enhance nurses’ adherence to evidence-based mechanical ventilation procedures. Nursing care alone, however, is not the only element that affects sickness severity as determined by the APACHE II grading system. Therefore, rather than establishing a direct causal relationship between the educational program and patient outcomes, APACHE II was included as an objective measure of sickness severity to compare the patient cohorts before and after the intervention.

Thus, this quasi-experimental study sought to compare APACHE II scores between the independent pre- and post-intervention patient cohorts and assess changes in the knowledge, professional competence, and clinical performance of intensive care unit nurses after engagement in a structured educational program.

## Methods

### Research design

The study employed a quasi-experimental single-group pretest-posttest design to investigate changes in the clinical performance, professional abilities, and knowledge of ICU nurses related to their involvement in a structured teaching program for treating patients on mechanical ventilation. Additionally, for mechanically ventilated patients admitted during the pre- and post-implementation periods, the Acute Physiology and Chronic Health Evaluation II (APACHE II) score was used to describe the severity of the illness, taking into account the fact that the two patient cohorts were independent and that APACHE II represents the initial severity within the first 24 h of ICU admission. This particular design was deemed suitable for assessing an educational intervention in an authentic clinical environment where randomization was impractical. Nonetheless, due to the absence of random assignment or a control group, caution is advised when drawing causal conclusions regarding patient outcomes. The study adhered to the guidelines outlined in the Transparent Reporting of Evaluations with Nonrandomized Designs (TREND) statement (24, 25).

### Setting

The research was carried out from March 2025 to September 2025 within the adult intensive care units of five governmental healthcare facilities situated in Port Said Governorate, Egypt. These establishments offer inclusive critical care services catering to patients with diverse medical and surgical conditions necessitating sophisticated life-support techniques, such as invasive mechanical ventilation. The involved hospitals comprised: – Al-Salam Port Said Hospital, featuring two intensive care units with a collective capacity of 21 ICU beds and a staffing of 34 ICU nurses. – Al Shifa Medical Complex (Unit A), encompassing three intensive care units housing 17 ICU beds and employing 35 ICU nurses. – El-Hayat Port Fouad Hospital, hosting one intensive care unit with 9 ICU beds and 19 ICU nurses. – Al Shifa Medical Complex (Unit B), comprising two intensive care units accommodating 17 ICU beds and 19 ICU nurses. – Al-Zahoor Central Hospital, with one intensive care unit comprising 10 ICU beds and 22 ICU nurses. All the included ICUs adhered to uniform evidence-based guidelines for the management of mechanical ventilation and holistic critical care. The ICU nursing staff operated in cooperation with intensivists, respiratory therapists, pharmacists, physiotherapists, and other medical professionals to deliver comprehensive care for critically unwell patients. The educational initiative specifically focused on enhancing the nursing competencies within this collaborative care framework.

### Subjects and population

The research encompassed two distinct cohorts: intensive care unit (ICU) nurses who engaged in an educational initiative and adult patients receiving mechanical ventilation whose Acute Physiology and Chronic Health Evaluation II (APACHE II) scores were evaluated pre and post the program’s implementation.

Regarding the ICU Nurses, a representative sample of 60 registered ICU nurses was selected in proportion from the involved medical facilities. Inclusion criteria encompassed being registered nurses in adult ICU settings, directly caring for mechanically ventilated patients, possessing a minimum of 6 months of ICU practice, and consenting to partake in the research. Conversely, exclusion criteria involved interns, nurses in orientation, individuals with less than 6 months of ICU experience, those exclusively in administrative roles, and those declining involvement.

Concerning the Mechanically Ventilated Patients, a deliberate selection of 50 adult patients under mechanical ventilation was chosen for APACHE II assessment. Patients admitted prior to and following the intervention were distinct, rendering the patient groups independent. APACHE II scores were computed for each patient within the initial 24 h of ICU admission in accordance with the original APACHE II scoring methodology. Inclusion criteria stipulated patients being 18 years or older, admitted to the designated ICUs, necessitating invasive mechanical ventilation for a minimum of 24 h, and having the requisite complete physiological and laboratory data for APACHE II score computation. Conversely, exclusion criteria involved individuals under 18 years, those transferred from another ICU after the initial 24 h of admission, patients lacking essential clinical data for APACHE II calculation, and those readmitted to the ICU during the same hospital stay.

### Sampling technique

The research used a convenience sampling strategy to recruit skilled ICU nurses from the participating medical institutions, ensuring that the sample mirrored the composition of ICU nursing staff within each hospital (26–29). A systematic process was followed to include mechanically ventilated patients meeting specified criteria, resulting in the formation of two distinct groups—a cohort of 50 patients before the intervention and a separate cohort of 50 patients after the intervention. The severity of illness was evaluated using the APACHE II scoring system to delineate the initial clinical status across both patient groups.

### Measures to minimize bias

In order to uphold methodological coherence, a standardized set of protocols was enacted consistently throughout the duration of the research. The educational intervention adhered to a unified procedure that was uniformly implemented across all medical facilities involved in the study. Consistency was rigorously maintained in various aspects, including the educational syllabus, instructional materials, teaching methodologies, training timetables, and program duration at each designated site. Oversight of all educational sessions was conducted by the principal investigator to ensure uniformity. Standardized tools for data collection and assessment were utilized for both pre- and post-intervention appraisals among all enrolled nurses. The evaluation of clinical performance involved the use of a standardized observational checklist under similar operational circumstances. Patient acuity levels were gauged utilizing the APACHE II scoring system, employing the original scoring criteria within the initial 24 h of ICU admission for all mechanically ventilated patients encompassed in both study phases. Nevertheless, certain clinical variables, such as primary diagnosis, comorbidities, baseline disease severity, ventilator configurations, and concurrent medical interventions, may remain beyond complete control within authentic ICU settings.

### Study tools

Four tools were used in this study:

*Tool (I):* nurses’ knowledge questionnaire: This tool consisted of a structured multiple-choice and list pre/post-test questionnaire. This tool was developed by the researcher after reviewing related literature (30–34) to assess the knowledge level of critical care nurses regarding the care of patients on mechanical ventilators. The questionnaire was developed specifically for this study, and the English version of the tool is provided as Supplementary File 1.

This tool includes two parts:

*Part (1):* In addition to some selected demographic data for nurses, such as age, marital status, qualification, years of experience, and previous training program.

*Part (2):* nurses’ knowledge regarding the care of patients on mechanical ventilators. It consisted of 73 items divided into six domains: anatomy and physiology of the respiratory system (12 questions), knowledge regarding mechanical ventilation (19 questions), mechanical ventilator modes, alarms, settings, complications, and nursing care and weaning from the mechanical ventilator (22 questions). Each item underwent evaluation utilizing a prescribed model answer key devised by the investigator. Accuracy of responses corresponded to a score of one (1), while inaccuracies were rated as zero (0). Cumulative scores for items within distinct domains were amalgamated to derive the domain-specific score, with the aggregate knowledge score computed by summing the scores of all items. The potential total score spanned from 0 to 73 points, with elevated scores denoting an augmented degree of nurses’ proficiency in the care of mechanical ventilation Supplementary File 1.

*Tool (II):* A short version of the nurse professional competence scale for measuring nurses’ self-reported competence (NPC Scale-SF) was developed by Nilsson et al. (35). Nursing Care (5 items), Value-Based Nursing Care (5 items), Medical and Technical Care (6 items), Care Pedagogics (5 items), Documentation and Administration of Nursing Care (8 items), and Development, Leadership, and Organization of Nursing Care (6 items) comprise the 35 items that make up the NPC Scale-SF. A 4-point Likert scale, with 1 denoting a very low degree and 4 denoting a very high degree, was used by participants to rate each item. While the total competence score was determined by adding up all 35 items, which resulted in a possible score ranging from 35 to 140, domain scores were determined by adding up the answers to the items within each domain. Higher self-reported levels of professional nursing competence were reflected by higher scores.

*Tool (III):* nurses’ practice observation checklist.

Following a comprehensive literature review (30, 33, 36–40), the investigator formulated an observational checklist detailed in Supplementary File 2 to evaluate the clinical performance of nurses tending to mechanically ventilated patients. The checklist encompassed five distinct proficiency areas: Hand Hygiene (consisting of 7 steps), Endotracheal Tube Suctioning (comprising 44 steps), Endotracheal Tube care (encompassing 39 steps), Management of Weaning from Mechanical Ventilation (with 97 steps), and Arterial Blood Gas (ABG) Analysis (comprising 49 steps), totaling 236 discrete observable actions. Observations of each step were executed and rated utilizing a binary scale: a score of 1 denoted correct execution, while 0 signified incorrect execution or omission. Scores for individual competencies were aggregated by summing the scores of their respective steps, and the cumulative performance score was derived by summing the scores across the five skill domains, with a potential score range of 0 to 236.

*Tool (IV):* patients’ demographic and clinical data collection sheet.

*Part I:* a data collection instrument designed by the researcher after a thorough examination of pertinent literature, intended for capturing demographic and clinical details of patients. It encompasses a range of information such as demographic particulars (gender, marital status, and employment status), clinical attributes (main diagnosis and coexisting conditions), specifics of mechanical ventilation (ventilator settings, fraction of inspired oxygen [FiO₂], positive end-expiratory pressure [PEEP], and tidal volume), and medical interventions like broad-spectrum antibiotics, vasopressors, sedatives, and neuromuscular blocking agents. Patient data, both demographic and clinical, were obtained from medical records and ICU files utilizing a standardized data collection template. Part II: The APACHE II score is a widely used system for assessing disease severity and predicting outcomes in ICU patients. The APACHE II score was used to assess disease severity among mechanically ventilated patients admitted during the pre-intervention period and those admitted during the post-intervention period (two independent patient groups). It was first introduced in 1981 and revised in 1985. The APACHE II score is calculated using a formula that includes 12 different physiologic measurements, the patient’s age, and their chronic health status. These measurements are taken within the first 24 h of a patient’s ICU admission. The components of the score are as follows: Acute Physiology Score (APS), which is derived from 12 clinical measurements, including vital signs, oxygenation, and lab values. Age points, with higher points for older patients. Points for chronic health conditions, such as severe organ insufficiency or an immunocompromised state. The total score can range from 0 to 71, with higher scores indicating a higher risk of mortality. The specific formula involves assigning a certain number of points to each measurement based on its deviation from normal values (41).

### Study procedure

The study was conducted in three phases (the preparatory phase, the implementation phase, and the evaluation phase).

#### Preparatory phase

In this phase, the researcher prepared data collection tools based on relevant literature. Nurses’ learning needs were assessed using three tools designed to evaluate their knowledge, competence, and practice in clinical settings. The same instruments were used for evaluation. Patient outcomes were assessed using the APACHE score. Additionally, the researcher assessed available places, time, equipment, supplies, and instructional materials for the training program.

##### Tools content validity and reliability

The validity and reliability of the study instruments were established and reported according to contemporary psychometric evaluation principles (35, 42). The study instruments’ content validity was ascertained through assessment by a panel comprising ten experts. This panel encompassed three proficient critical care physicians specializing in intensivist, anesthesiology, and pulmonology, along with extensive experience in managing mechanically ventilated patients. Additionally, it included three academic scholars in critical care nursing and mechanical ventilation research, three senior intensive care nurses occupying roles such as head nurses or clinical instructors, and one bilingual specialist overseeing the translation and back-translation procedures. The experts evaluated the instruments concerning their lucidity, pertinence, inclusiveness, and alignment with the study’s objectives. Subsequently, their feedback and suggestions were integrated, leading to the requisite modifications of the instruments before commencing data collection.

The Nurses’ Professional Competence Scale–Short Form (NPC Scale-SF) has been established as a valid assessment tool. Its original creators demonstrated sound internal consistency, as evidenced by Cronbach’s alpha coefficients ranging from 0.71 to 0.86 across the scale’s six competence domains. The scale’s construct validity was confirmed through exploratory factor analysis, revealing a six-factor model that accounted for 53.6% of the variance, supported by a robust Kaiser–Meyer–Olkin value of 0.920, indicating high sampling adequacy. The Arabic adaptation of the NPC Scale-SF underwent meticulous forward and backward translation by a bilingual expert proficient in both English and Arabic to ensure linguistic and conceptual fidelity.

A preliminary investigation was undertaken on a subset comprising 10% of the total study population to assess the lucidity, viability, and relevance of the research tools, as well as to determine the time needed for data gathering. After the pilot study, no significant alterations were deemed necessary. The subjects partaking in the pilot phase were omitted from the final analysis and subsequently substituted with a different cohort of nurses. The internal consistency reliability of the measurement instruments devised by the researcher was evaluated utilizing Cronbach’s alpha coefficient. The knowledge assessment survey exhibited a commendable level of reliability (Cronbach’s *α* = 0.903), the observational checklist manifested a satisfactory reliability level (Cronbach’s α = 0.890), and the Arabic iteration of the NPC Scale-SF showcased a favorable level of internal consistency within the current investigation (Cronbach’s α = 0.894).

Regarding the data collection form pertaining to patient demographic and clinical information, the validity of its content was ascertained through appraisal by an additional committee of five specialists in the fields of Medical-Surgical Nursing and Critical Care Medicine. These experts evaluated the instrument’s clarity, relevance, comprehensiveness, and alignment with the research objectives. After their review, the data collection tool underwent revisions in accordance with their suggestions before being put into practice. The incorporation of the APACHE II scoring system in the latter section of the instrument is noteworthy as it is a globally recognized and thoroughly validated tool for evaluating disease severity and prognosticating mortality rates among critically ill patients. Given the standardized nature of the APACHE II scoring system, its well-established psychometric properties have been extensively documented in prior research and were embraced in the current study.

##### Ethical consideration

Ethical approval code NUR (4/1/2024) (33) was obtained from the institutional review board at Port Said University, with voluntary participation, anonymity assurance, and freedom to withdraw from the study at any time, with no penalty resulting from this withdrawal. Before data collection, written informed consent was obtained from all participants after they were provided with a clear explanation of the study’s purpose, procedures, risks, and benefits.

##### Development of the educational program

The researcher developed the educational program after reviewing the previously mentioned literature and based on the nurses’ identified needs from the pretest. The following steps were adopted to develop the program, and the program’s general and specific objectives were stated.

Planning the program: the content of the program was arranged into seven sessions in addition to the preliminary one. The content of the program covered two parts related to data collection tools, knowledge, and practice of the procedures regarding the care of patients on mechanical ventilators.

#### Implementation phase

The researcher began data gathering immediately after receiving authorization. Data were collected in the existing environment. The principal investigator introduced herself at the start of each interview. Five groups of nine to sixteen nurses were formed, meeting during their shifts, between 12:30 and 1:15 p.m. for the early shift and 2:30 and 3:15 p.m. for the late shift. Over 6 weeks, each group had one 45-min training session per week. Before starting, each group received an explanation of the training program’s plan, learning objectives, and significance, along with a handout. The curriculum was delivered using lectures, labs, re-demonstrations, and multimedia materials to ensure clarity and understanding. Each session began with a quick review of previous material, followed by a summary of the current session’s goals. Nurses’ knowledge and competence were assessed before and after the program using tools I and II. For the practical portion, assessed with tool III, nurses observed the researcher demonstrating patient care procedures on mechanical ventilators, then repeated the procedure themselves for assessment. The researcher explained each step’s reasoning and safety measures and addressed any unclear steps before proceeding. Nurses were asked to self-assess their performance, with the researcher emphasizing that errors were part of the learning process. Immediate feedback was given after re-demonstrations, and nurses were encouraged to reflect on their performance. Each nurse practiced the procedure multiple times under guidance until proficient. Finally, the researcher provided constructive feedback and corrected any errors. After the educational program was introduced, a separate cohort of mechanically ventilated patients admitted post-intervention had their APACHE II scores computed. Each patient’s APACHE II score was assessed within 24 h of ICU admission following the standardized scoring method used before the intervention. As the patients from pre- and post-intervention phases were distinct cohorts, no patient matching occurred. The APACHE II results served to evaluate the severity of patients across the two-time frames.

#### Evaluation phase

In the context of program outcome assessment, the researcher delineated to nurses that her function was confined to the observation and recording of standard clinical activities linked to engagement in the educational intervention. Notwithstanding the observation of clinical procedures, nurses were not apprised of any probable connection to patient-specific metrics. The exclusive use of the APACHE II score was to delineate the severity of illness in ICU patients admitted before and after the intervention, rather than to assess the efficacy of the program. Each nurse involved in the study was evaluated three times immediately following the program’s implementation, using tools I, II, and III. Following this, the average was determined.

### Statistical analysis

The statistical analysis of the data was performed using IBM SPSS software version 27.0 (Armonk, NY: IBM Corp, released 2020). Categorical data were summarized as numbers and percentages. For continuous data, normality was assessed using the Kolmogorov–Smirnov test. Quantitative data were described using range (minimum and maximum), mean, and standard deviation. Pre- and post-intervention scores were compared using the paired-samples *t*-test, whereas the independent-samples *t*-test was used to compare the mean scores between Group 1 and Group 2 patients. The magnitude of the intervention effect was estimated by calculating the mean difference (MD) with its 95% confidence interval (95% CI) and Cohen’s d effect size.

Univariate linear regression was first performed to examine the association between each predictor and the APACHE II score. A multivariable linear regression model was then constructed to identify independent predictors after adjustment for potential confounders. The assumptions of linear regression were verified before model interpretation. Residual independence was assessed using the Durbin–Watson statistic (2.199), indicating no autocorrelation. Multicollinearity was evaluated by the variance inflation factor (VIF; 1.096–1.461) and pairwise correlation coefficients (<0.70), confirming that multicollinearity was not a concern. Regression results are presented as unstandardized regression coefficients (B) with 95% confidence intervals. Model fit was assessed using the F statistic and the coefficient of determination (*R*^2^). A two-sided *p*-value ≤0.05 was considered statistically significant.

## Results

Table 1 indicates that most of the nurses studied (56.7%) were under 30 years of age. A significant majority of the nurses (83.3%) were married. The largest proportion of nurses (36.7%) held a Bachelor of Nursing degree, while equal percentages (31.7% each) held a Nursing Diploma and a Nursing Technician Institute qualification. Nearly half of the nurses (46.7%) had less than 5 years of experience caring for patients on ventilators. A significant majority of the nurses (76.7%) had previously studied nursing care for patients on ventilators.

**Table 1:** Distribution of the nurses studied according to demographic data (*n* = 60).

Demographic data	*n*	%
Age (years)		
<30	34	56.7
30– < 35	14	23.3
≥35	12	20.0
Mean ± SD.	**29.78 ± 4.59**	
Marital status		
Married	50	83.3
Unmarried	10	16.7
Educational level		
Nursing Diploma	19	31.7
Nursing Technician Institute	19	31.7
Bachelor of Nursing	22	36.7
Years of experience in nursing		
<5	16	26.7
5– < 10	25	41.7
10– < 20	12	20.0
≥20	7	11.7
Mean ± SD.	8.98 ± 6.81	
Years of experience in dealing with patients on a ventilator		
<5	28	46.7
5– < 10	25	41.7
10– < 20	7	11.7
Mean ± SD.	**5.30 ± 3.92**	
Have you studied the nursing care for a patient with a ventilator?		
No	14	23.3
Yes	46	76.7
Are written guidelines available in the unit for ventilator care?		
No	7	11.7
Yes	53	88.3

Bold values indicate the mean ± standard deviation (SD).

Table 2 illustrates a significant increase in knowledge scores post-training compared to pre-training across all domains assessed, as evidenced by high *t*-values and *p*-values below 0.001. The narrow confidence intervals and substantial Cohen’s d values (e.g., Weaning from mechanical ventilator d = 2.395) suggest consistent score improvements after the educational program. The study demonstrates enhanced immediate knowledge following program participation, without implying causation in the one-group pre-post design.

**Table 2:** Differences in studied nurses’ knowledge throughout an educational program regarding care to mechanically ventilated patients (*n* = 60).

Knowledge	Pre	Post	*t*	*p*	MD (95% CI)	Cohen d
Anatomy and physiology of the respiratory system (0–12)						
Min. – Max.	8.0–12.0	10.0–12.0	7.425*	<0.001*	1.366 (0.998–1.734)	0.959
Mean ± SD.	9.70 ± 1.15	11.07 ± 0.71				
Mechanical ventilation (0–19)						
Min. – Max.	10.0–15.0	14.0–19.0	8.307*	<0.001*	1.933 (1.468–2.399)	1.072
Mean ± SD.	12.78 ± 1.50	14.72 ± 1.38				
Mechanical ventilator, modes, alarms, settings, complications and nursing care (0–20)						
Min. – Max.	2.0–10.0	5.0–11.0	7.655*	<0.001*	2.683 (1.982–3.385)	0.988
Mean ± SD.	5.53 ± 2.46	8.22 ± 1.67				
Weaning from the mechanical ventilator (0–22)						
Min. – Max.	6.0–12.0	14.0–18.0	18.549*	<0.001*	6.283 (5.606–6.961)	2.395
Mean ± SD.	9.63 ± 2.10	15.92 ± 1.25				
Overall knowledge (0–73)						
Min. – Max.	30.0–45.0	47.0–58.0	17.016*	<0.001*	12.267 (10.824–13.709)	2.197
Mean ± SD.	37.65 ± 4.94	49.92 ± 3.04				

SD, standard deviation; *t*, paired *t*-test; *: *p* < 0.001 indicates a statistically significant difference between pre-test and post-test scores.

The study reveals a consistent increase in post-training scores compared to pre-training scores across all domains of the Nurse Professional Competence-Short Version Scale (Table 3). Significantly large differences were evident between the two assessment points, supported by high *t*-values and *p*-values below 0.001. The data indicates stable and substantial score enhancements post-participation in the educational program, notably in nursing care, value-based care, medical and technical care, care pedagogics, documentation and administration, and leadership/organization domains, with notable effect sizes (Cohen’s d) ranging from 4.764 to 7.744. The post-training scores, particularly in overall competence, were notably higher than baseline, denoting immediate competence improvements post-program completion. The results suggest an association between program participation and heightened professional competence scores without inferring direct causation, in line with the one-group pre-post design.

**Table 3:** Differences in studied nurses’ competence throughout an educational program regarding care to mechanically ventilated patients (*n* = 60).

Nurse Professional Competence-Short Version Scale (NPC-SF)	Pre	Post	*t*	*p*	MD (95% CI)	Cohen d
Nursing care (5–20)						
Min. – Max.	5.0–10.0	18.0–20.0	42.940*	<0.001*	12.183 (11.616–12.751)	5.543
Mean ± SD.	7.63 ± 2.17	19.82 ± 0.43				
Value-based nursing care (5–20)						
Min. – Max.	5.0–10.0	19.0–20.0	46.073*	<0.001*	11.600 (11.096–12.104)	5.948
Mean ± SD.	8.35 ± 1.91	19.95 ± 0.22				
Medical and technical care (6–24)						
Min. – Max.	6.0–13.0	21.0–24.0	36.898*	<0.001*	13.217 (12.500–13.933)	4.764
Mean ± SD.	10.43 ± 2.49	23.65 ± 0.68				
Care pedagogics (5–20)						
Min. – Max.	5.0–10.0	19.0–20.0	50.898*	<0.001*	11.050 (10.616–11.484)	6.571
Mean ± SD.	8.88 ± 1.62	19.93 ± 0.25				
Documentation and administration of nursing care (8–32)						
Min. – Max.	12.0–18.0	28.0–32.0	56.677*	<0.001*	16.400 (15.821–16.979)	7.317
Mean ± SD.	14.83 ± 1.67	31.23 ± 1.28				
Development, leadership, and organization of nursing care (6–24)						
Min. – Max.	8.0–12.0	22.0–24.0	51.823*	<0.001*	12.700 (12.210–13.190)	6.690
Mean ± SD.	10.77 ± 1.44	23.47 ± 0.81				
Overall competence (35–140)						
Min. – Max.	46.0–70.0	132.0–140.0	59.986*	<0.001*	77.150 (74.576–79.724)	7.744
Mean ± SD.	60.90 ± 8.44	138.05 ± 2.75				

SD, standard deviation; *t*, paired *t*-test; *: *p* < 0.001 indicates a statistically significant difference between pre-test and post-test scores.

The research outcomes demonstrate a consistent improvement in post-training assessment scores compared to pre-training scores across various clinical procedures (Table 4). Statistical analysis revealed significant differences between the two assessment points, with highly significant t values and *p*-values below 0.001 in all domains. Noteworthy mean differences, along with precise confidence intervals, indicate substantial and enduring score enhancements post-program completion. Enhanced post-training scores were particularly prominent in specific procedures, including hand hygiene, suctioning of the endotracheal tube, endotracheal tube care, patient care during ventilator weaning, and arterial blood gas procedures. Effect sizes (Cohen’s d) suggested significant discrepancies between pre- and post-training assessments, with the most pronounced difference observed in the overall practice score, showing markedly increased post-training values compared to baseline levels. These results align with the specialized findings, indicating an immediate post-training improvement in practice scores across all evaluated competencies. The findings suggest a positive relationship between program participation and improved post-training clinical practice scores, although they do not establish a direct causal link, in line with the methodology of the one-group pre-post study.

**Table 4:** Differences in studied nurses’ practice throughout an educational program regarding care to mechanically ventilated patients (*n* = 60).

Practice	Pre	Post	*t*	*p*	MD (95% CI)	Cohen d
Hand washing (0–7)						
Min. – Max.	0.0–4.0	6.0–7.0	21.387*	<0.001*	4.583 (4.155–5.012)	2.761
Mean ± SD.	2.40 ± 1.64	6.98 ± 0.13				
Suctioning of the endotracheal tube (0–44)						
Min. – Max.	17.0–30.0	40.0–44.0	23.247*	<0.001*	17.167 (15.689–18.644)	3.001
Mean ± SD.	25.20 ± 4.66	42.37 ± 1.54				
Care of the endotracheal tube (0–39)						
Min. – Max.	8.0–14.0	36.0–39.0	62.161*	<0.001*	27.183 (26.308–28.058)	8.025
Mean ± SD.	10.80 ± 2.66	37.98 ± 1.11				
Providing care to patients during weaning from mechanical ventilation (0–97)						
Min. – Max.	32.0–40.0	85.0–97.0	104.431*	<0.001*	59.200 (58.066–60.334)	13.482
Mean ± SD.	35.80 ± 3.28	95.0 ± 2.59				
ABG (0–49)						
Min. – Max.	13.0–31.0	46.0–49.0	28.051*	<0.001*	25.550 (23.727–27.373)	3.621
Mean ± SD.	22.80 ± 7.34	48.35 ± 1.25				
Overall practice (0–236)						
Min. – Max.	89.0–106.0	214.0–236.0	117.697*	<0.001*	133.683 (131.411–135.956)	15.195
Mean ± SD.	97.0 ± 5.60	230.68 ± 6.07				

SD, standard deviation; *t*, paired *t*-test; *: *p* < 0.001 indicates a statistically significant difference between pre-test and post-test scores.

Comparison of the socio-demographic characteristics between the pre- and post-intervention patient cohorts (Table 5). No statistically significant differences were observed in sex, marital status, or employment status between the two cohorts (*p* > 0.05).

**Table 5:** Comparison of socio-demographic characteristics between the pre- and post-intervention patient cohorts.

Demographic characteristics	Pre (*n* = 50)		Post (*n* = 50)		χ2	*p*
	No.	%	No.	%		
Age (years)						
<44	20	40.0	15	30.0	5.799	0.215
45–54	5	10.0	5	10.0		
55–64	9	18.0	10	20.0		
65–74	16	32.0	15	30.0		
>74	0	0.0	5	10.0		
Sex						
Male	41	82.0	44	88.0	0.706	0.401
Female	9	18.0	6	12.0		
Marital status						
Married	37	74.0	43	86.0	2.250	0.134
Unmarried	13	26.0	7	14.0		
Employment status						
Employed	28	56.0	21	42.0	1.961	0.161
Unemployed/Retired	22	44.0	29	58.0		

χ2: Chi-square test. *p*: *p*-value for comparing the two studied groups.

Comparison of the clinical characteristics, mechanical ventilation parameters, and medical treatments between the pre- and post-intervention patient cohorts (Table 6). No statistically significant differences were observed in the measured clinical characteristics, ventilator parameters, or treatment variables (all *p* > 0.05).

**Table 6:** Comparison of clinical characteristics, mechanical ventilation parameters, and medical treatments in the pre- and post-intervention patient cohorts.

Clinical characteristics	Pre (*n* = 50) No. (%)	Post (*n* = 50) No. (%)	χ^2^	*p*
Primary diagnosis				
Pneumonia/ARDS	25 (50.0)	23 (46.0)	3.010	0.556
Sepsis/Septic shock	5 (10.0)	11 (22.0)		
COPD exacerbation	9 (18.0)	6 (12.0)		
Post-operative complications	7 (14.0)	6 (12.0)		
Cardiac failure	4 (8.0)	4 (8.0)		
Comorbidities (more than one answer may be selected)				
Hypertension	19 (38.0)	19 (38.0)	0.000	1.000
Diabetes Mellitus	11 (22.0)	13 (26.0)	0.219	0.640
Chronic kidney disease	9 (18.0)	11 (22.0)	0.250	0.617
Ischemic heart disease	8 (16.0)	6 (12.0)	0.332	0.564
No comorbidities	3 (6.0)	1 (2.0)	1.042	0.307
Other	19 (38.0)	19 (38.0)	–	–
Mechanical ventilation				
*Ventilator mode*				
Assist-Control (A/C)	29 (58.0)	36 (72.0)	2.210	0.331
SIMV	17 (34.0)	11 (22.0)		
Pressure Support Ventilation (PSV)	4 (8.0)	3 (6.0)		
*Fraction of Inspired Oxygen (FiO₂)*				
<60%	28 (56.0)	33 (66.0)	1.051	0.305
≥60%	22 (44.0)	17 (34.0)		
Positive End-Expiratory Pressure (PEEP)				
<8 cm H₂O	32 (64.0)	36 (72.0)	0.735	0.391
≥8 cm H₂O	18 (36.0)	14 (28.0)		
*Tidal volume*				
<6 mL/kg	34 (68.0)	38 (76.0)	2.485	0.334
6–8 mL/kg	14 (28.0)	8 (16.0)		
>8 ml/kg	2 (4.0)	4 (8.0)		
Medical treatment				
*Broad-spectrum antibiotics*				
Yes	27 (54.0)	33 (66.0)	1.500	0.221
No	23 (46.0)	17 (34.0)		
*Vasopressors*				
Yes	17 (34.0)	14 (28.0)	0.421	0.517
No	33 (66.0)	36 (72.0)		
Sedation				
Yes	44 (88.0)	46 (92.0)	0.444	0.505
No	6 (12.0)	4 (8.0)		
Neuromuscular blockers				
Yes	10 (20.0)	11 (22.0)	0.060	0.806
No	40 (80.0)	39 (78.0)		

χ2, Chi-square test; *p*, *p*-value for comparing the two studied groups.

Comparison of APACHE II scores between the pre- and post-intervention patient cohorts (Table 7). A statistically significant difference in APACHE II scores was observed between the two independent cohorts (*t* = 4.366, *p* < 0.001), with a mean difference of 8.12 (95% CI, 4.43–11.81) and a large effect size (Cohen’s d = 0.873).

**Table 7:** Comparison of illness severity between the pre- and post-intervention patient cohorts (*n* = 50).

Total APACHE II Score	Pre	Post	*t*	*p*	MD (95% CI)	Cohen d
Min. – Max.	23.0–49.0	15.0–49.0	4.366^*^	<0.001^*^	8.120 (4.429–11.810)	0.873
Mean ± SD.	35.42 ± 7.52	27.30 ± 10.79				

SD, standard deviation; *t*, student *t*-test. *p*: *p*-value for comparing the two studied groups. *: Statistically significant at *p* ≤ 0.05 (All tests were two-tailed).

The univariate and multivariable linear regression analyses of the variables related to APACHE II scores are shown in Table 8. APACHE II scores were considerably lower in the post-intervention cohort compared to the pre-intervention cohort in the univariate analysis (B = −8.120, 95% CI: −11.811 to −4.429, *p* < 0.001). This link remained significant after controlling for the model’s variables (B = −9.089, 95% CI: −11.919 to −6.260, *p* < 0.001). Higher APACHE II scores were also independently linked to older age and the presence of comorbidities (*p* < 0.05). The final model explained 62.6% of the variance in APACHE II scores (*R*^2^ = 0.626; adjusted *R*^2^ = 0.570) and was statistically significant (*F* = 11.090, *p* < 0.001). These results, however, suggest an association rather than a causal effect of the training program on illness severity because of the quasi-experimental design and the use of two separate patient cohorts.

**Table 8:** Factors associated with APACHE II score using univariate and multivariable linear regression.

Variables	Univariate		Multivariable	
	*p*	B (LL – UL 95%C. I)	*p*	B (LL – UL 95%C. I)
Groups				
Pre (Ref.)				
Post	<0.001*	−8.120 (−11.811 – −4.429)	<0.001*	−9.089 (−11.919 – −6.260)
Age (years)				
<44 (Ref.)				
45–54	0.015*	7.043 (1.407–12.679)	<0.001*	9.195 (4.175–14.216)
55–64	0.364	2.059 (−2.421–6.538)	0.044*	4.059 (0.114–8.003)
65–74	<0.001*	15.162 (11.285–19.039)	<0.001*	16.922 (13.435–20.409)
>74	0.108	6.143 (−1.372–13.658)	0.001*	12.232 (5.308–19.155)
Sex [Male ref.]				
Female	0.516	1.851 (−3.785–7.487)	0.207	−2.686 (−6.885–1.513)
Primary diagnosis				
Pneumonia/ARDS	0.917	−0.212 (−4.248–3.825)	0.233	−1.893 (−5.024–1.239)
Sepsis/Septic Shock	0.784	0.762 (−4.738–6.261)	0.803	−0.570 (−5.095–3.955)
*Comorbidities*	0.819	1.188 (−9.102–11.477)	0.037*	8.348 (0.523–16.173)
Treatment				
Broad-spectrum Antibiotics	0.943	−0.150 (−4.267–3.967)	0.484	1.093 (−2.001–4.187)
Vasopressors	0.583	1.208 (−3.146–5.562)	0.918	0.160 (−2.915–3.236)
Sedation	0.906	0.400 (−6.323–7.123)	0.519	1.580 (−3.272–6.432)
Neuromuscular Blockers	0.567	1.413 (−3.531–6.357)	0.677	−0.791 (−4.559–2.976)
Calculations			*F* = 11.090*, *p* < 0.001*	
			R2 = 0.626, adjusted R2 = 0.570	

B, unstandardized coefficients; *F*, *p*: *f* and *p* values for the model; C. I, confidence interval. LL: Lower limit, UL: Upper Limit, *R*^2^: Coefficient of determination. *: Statistically significant at *p* ≤ 0.05 (All tests were two-tailed). Primary diagnosis (reference = Other diagnoses).

## Discussion

The current study assessed how ICU nurses’ clinical practice, professional competence, and knowledge changed after taking part in a structured mechanical ventilation management education program. To provide safe, evidence-based care, ICU nurses need to possess current theoretical knowledge and practical skills due to the intricacy of mechanical ventilation. Poor adherence to evidence-based ventilator care procedures has been linked to avoidable consequences, such as ventilator-associated pneumonia, according to earlier research, underscoring the significance of ICU nurses’ ongoing professional development (43–45). Higher post-training ratings were found in the current study in all evaluated domains of clinical practice, professional competence, and knowledge. These results show that taking part in the organized educational program was linked to improved performance right after training. However, these results should be viewed as an association rather than proof of a causal effect and should not be seen as proof of a direct impact on patient outcomes due to the quasi-experimental methodology and lack of a control group.

Intensive care unit (ICU) nurses who participated in the educational program had considerably higher post-training knowledge scores, according to the current study. Higher scores were found, specifically in the area of knowledge about the care of patients on mechanical ventilation. These results imply that better short-term knowledge across the evaluated domains was linked to involvement in the organized educational program. Similar results have been documented in earlier research assessing evidence-based teaching methods that enhance nurses’ understanding of mechanical ventilation and evidence-based care, such as interactive instruction, simulation-based learning, reflective practice, and experiential training (5, 46–50). Improvements in knowledge do not always result in long-term professional competence or clinical practice, even though improved knowledge scores are a significant immediate educational benefit. Long-term information retention could not be ascertained because knowledge was evaluated right after the educational program was finished. Periodic refresher training, structured feedback, and ongoing professional development are advised to support knowledge retention over time in accordance with prior educational studies. To assess the sustainability of knowledge gains and their possible correlation with later changes in professional competence, clinical practice, and patient outcomes, future longitudinal studies with repeated follow-up assessments are required.

Also, Intensive care unit (ICU) nurses who participated in the educational program had higher immediate post-training professional competency levels, according to the current study. The proper care of patients on mechanical ventilation requires professional competence, which encompasses theoretical knowledge, clinical judgment, technical skills, communication, and decision-making. Following involvement in the educational program, higher competency scores were noted in all of these domains (51–55). In line with earlier research assessing simulation-based learning, reflective practice, and competency-based educational approaches in critical care nursing, these results imply that involvement in the structured educational program was linked to higher perceived competence (56). Higher competency scores, however, should not be seen as proof of better patient outcomes or clinical performance. Competence was evaluated right after the instructional session was finished, and the current study did not look at whether greater competence ratings resulted in improvements in bedside practice or patient outcomes. As a result, it is still unclear if these findings will be sustainable in the long run. In order to sustain competence over time, prior research has highlighted the significance of ongoing professional development, refresher training, and structured feedback. In a similar vein, Norouzrajabi et al. (57) found that ICU nurses who received organized educational interventions had higher competency scores. To determine whether greater competence scores are sustained over time and whether they are linked to later modifications in clinical practice and patient outcomes, more longitudinal studies with longer follow-up and objective assessments of clinical performance are required.

Furthermore, ICU nurses who participated in the educational program had higher immediate post-training clinical practice scores, according to the current study. Increased adherence to recommended clinical procedures at the time of evaluation was linked to program participation, as evidenced by higher scores across all areas of mechanical ventilation care. These results are in line with earlier research showing that skill-based training, blended learning strategies, and structured educational interventions were linked to increased adherence to evidence-based mechanical ventilation procedures, such as airway suctioning and endotracheal tube care (5, 58–61). These results show significant short-term educational benefits, but they should not be seen as proof of better patient outcomes. Therefore, rather than indicating improvements in patient outcomes, the results should be viewed as higher immediate post-training clinical practice scores. Mogyoródi et al. (62) reported similar results, observing increased adherence to ventilator care standards after focused instructional interventions. It takes continual professional growth, recurring refresher training, and organizational support to sustain these achievements. Furthermore, the long-term sustainability of these results is still unknown because clinical practice was evaluated right after the educational session was finished (63, 64). Lastly, the likelihood of a ceiling effect was raised by post-intervention scores that were close to the upper bounds of multiple evaluation scales. Future research should take into account longer follow-up periods and more discriminative evaluation techniques to better identify performance at higher competency levels, even though this was not officially tested.

Moreover, the current study showed that the post-intervention patient cohort had lower APACHE II scores than the pre-intervention cohort; nevertheless, this difference should be interpreted cautiously. The observed decrease in sickness severity may be due to differences in case mix or unmeasured clinical features rather than the direct effect of the training program because the study included two separate patient cohorts and did not completely account for potential confounding factors. This interpretation is consistent with earlier research (62, 64–66) showing that APACHE II is not intended as a gauge of the efficacy of educational interventions but rather as a predictive indicator of illness severity within the first 24 h of ICU admission. As a result, even though APACHE II offers an objective evaluation of physiological disturbances, it should not be utilized to conclude the causal relationship between patient outcomes and educational initiatives. These factors emphasize how crucial it is to interpret the results within the study’s methodological limitations and emphasize the necessity of future research using more controlled designs and longer follow-up times to assess the actual influence of educational interventions on clinical outcomes. On the other hand, after structured clinical pathways—which are clinical rather than educational interventions—were implemented, Badawi et al. (67) found slight decreases in APACHE II scores. When considered collectively, the present results are consistent with previous research showing that APACHE II is not sensitive enough to detect changes brought about by educational interventions.

After accounting for the assessed factors in the model, the regression analyses showed that the post-intervention cohort had lower APACHE II scores. However, this link should not be construed as proof that the educational program affected the severity of sickness because the patient cohorts were independent and residual confounding from unmeasured clinical factors cannot be excluded. Differences between cohorts are more likely to reflect variations in case mix or other clinical characteristics than the impact of the educational intervention, as APACHE II predominantly represents patients’ physiological status during the first 24 h after ICU admission. The lack of significant correlations for diagnosis, comorbidities, and organ dysfunction in the current adjusted study contrasts with earlier findings, despite prior research emphasizing the significance of these factors in influencing APACHE II scores. Rather than suggesting a lack of therapeutic importance for these variables, this divergence could be explained by sample characteristics, relative uniformity in case mix, or contextual variations between ICU settings (65–67).

The current study showed that nurses who took part in the educational program had better immediate post-training scores in clinical practice, knowledge, and abilities. The literature as a whole, however, offers a variety of conclusions; several studies have shown conflicting relationships between patient outcomes and educational interventions. For instance, Guilhermino et al. (64) found that while impacts on ICU mortality and duration of stay were unclear, nurse education on mechanical ventilation improved several process indicators. In a similar vein, Guilhermino et al. (64) and Branco et al. (68) discovered that while standardized or instructional treatments increased adherence to ventilator care protocols, patient-level results were not consistently improved. Shorter weaning times but inconsistent decreases in total breathing time were found in a recent quasi-experimental investigation by Xue et al. (15), indicating that patient characteristics and contextual factors may affect these correlations. When taken as a whole, these results emphasize the need for careful interpretation and show that the changes seen in this study are more likely to be short-term educational gains than conclusive proof of improved patient outcomes.

## Conclusion and recommendations

The findings of the present study suggest that engagement in a structured educational regimen was linked to heightened immediate post-training scores in the knowledge, professional competence, and clinical execution of ICU nurses concerning the care of mechanically ventilated patients. These results underscore the importance of continuous professional advancement in bolstering nurses’ readiness to deliver care grounded in evidence within intensive care environments. Nevertheless, due to the quasi-experimental nature of the study and the exclusive focus on short-term post-intervention results, it is advised not to construe the outcomes as definitive proof of a direct causal impact on patient clinical results. Healthcare establishments might contemplate integrating structured, evidence-based educational initiatives into regular professional growth initiatives to foster sustained competence enhancement. Routine review sessions, competency evaluations, and institutional backing could aid in maintaining enhancements in knowledge and clinical performance over time. Subsequent investigations should utilize randomized controlled trials or multi-center prospective methodologies with prolonged monitoring periods to explore enduring links between educational interventions and nursing efficacy. These inquiries should control for critical patient-related variables that could act as confounders—including underlying diagnosis, initial severity, coexisting conditions, ventilator configurations, and concurrent therapies—to better elucidate the association between nursing educational schemes and patient outcomes. Additionally, forthcoming explorations could investigate innovative educational strategies, such as simulation-driven learning and emerging digital tools, to gauge their efficacy in fortifying nursing education and clinical adeptness.

### Strengths and limitations

Several methodological strengths are highlighted in the study. First, it addressed a known educational deficit in critical care nursing by introducing a methodical teaching endeavor based on empirical research and customized to the intricate needs of caring for patients on mechanical ventilation. Second, all participating Intensive Care Units (ICUs) adhered to a uniform protocol for the intervention, guaranteeing uniformity in instructional materials, educational content, and evaluation techniques. Third, a thorough evaluation of immediate educational benefits was made possible by the use of validated instruments to assess many aspects of nursing performance, such as knowledge, skills, and clinical application. In order to contextualize the severity of the illness and describe changes in the case mix throughout the course of the study, patient demographic and clinical data were also methodically gathered. All things considered, the study adds to the expanding corpus of research on organized educational interventions in critical care and offers insightful information for developing future initiatives targeted at enhancing ventilator-related nursing competencies. Despite these advantages, there are a few drawbacks that need to be noted. In addition to being susceptible to testing effects, observer bias, the Hawthorne effect, and temporal changes unrelated to the intervention, the one-group pre-post design restricts causal inference. The lack of a control group makes it more difficult to ascertain whether program participation or outside factors were responsible for the benefits that were seen. Furthermore, the possibility of assessment bias is increased by the lead investigator’s dual role in training and evaluation and the absence of assessor blinding. Although multivariable regression adjusted for measured confounders, residual confounding cannot be excluded because of the quasi-experimental design. Furthermore, the distribution of patients among the participating hospitals was not analyzed separately, making it unable to assess differences in patient characteristics or clinical practices at the hospital level, which could lead to residual confounding. Since APACHE II is intended to evaluate the severity of illness during the first 24 h of ICU admission, fluctuations in scores are more likely to be the result of variances in the case mix than of the educational intervention. Moreover, results are mostly descriptive when sophisticated analytical techniques like multilevel modeling, mediation analysis, or time-adjusted methods are not used. Conclusions about long-term retention or sustainability are limited by the immediate post-training evaluation of knowledge, competence, and practice. Lastly, despite the inclusion of supportive literature, the existence of conflicting and neutral results in earlier research highlights the uneven conversion of educational interventions into better patient outcomes, highlighting the necessity of careful interpretation.

### Implications for nursing practice

This research highlights the significance of implementing structured, competency-based educational programs to enhance nurses’ competence in the management of mechanically ventilated patients. While advancements in APACHE II scores should be approached with caution due to differences in case intricacy, enhancing nurses’ skills is crucial for advancing safe clinical procedures, early identification of complications, and adherence to evidence-based ventilator care guidelines. Incorporating systematic education into regular ICU routines, backed by seasoned clinical mentors and ongoing organizational dedication, can promote ongoing professional growth and play a role in enhancing the general standard of critical care provision.

## Data Availability

The datasets used and/or analyzed during the current study are available from the corresponding author on reasonable request.
