# Supplementary material for: Enhancing nurses’ competence in mechanical ventilation: a quasi-experimental study and its association with patient severity using APACHE II
Source: Front Med (Lausanne). 2026 Aug 27;13:1891958. doi: 10.3389/fmed.2026.1891958 (PMC13587796; doi:10.3389/fmed.2026.1891958)
Supplement: Supplementary file 1 [file Data_Sheet_1.docx]

**Tool I: Nurses' knowledge questionnaire**

**Part I: Nurses' Demographic and Professional Characteristics**

**Instructions**

This questionnaire aims to assess nurses' knowledge regarding the nursing care of mechanically ventilated patients. It is not an examination of your knowledge but is intended to identify educational needs that may contribute to improving the quality of nursing care. Your responses will remain confidential and will be used solely for research purposes.

**A. Demographic Characteristics**

**Code Number:** __________________

**1. Age (years)**

□ Less than 30 years

□ 30 to less than 35 years

□ 35 years or older

**2. Marital Status**

□ Married

□ Unmarried

**3. Educational Level**

□ Nursing Diploma

□ Nursing Technical Institute

□ Bachelor of Nursing

**4. Years of Experience in Nursing**

□ Less than 5 years

□ 5 to less than 10 years

□ 10 to less than 20 years

□ 20 years or more

**5. Years of Experience in Caring for Patients on Mechanical Ventilation**

□ Less than 5 years

□ 5 to less than 10 years

□ 10 to less than 20 years

**6. Have You Studied Nursing Care for Patients Receiving Mechanical Ventilation?**

□ Yes

□ No

**7. Are Written Guidelines for the Nursing Care of Mechanically Ventilated Patients Available in Your Unit?**

□ Yes

□ No

**Part II: Nurses' Knowledge Regarding the Care of Mechanically Ventilated Patients**

**Section I. Anatomy and Physiology of the Respiratory System (12 Items)**

**Instructions:**
Please read each question carefully and select the **one best answer** by marking (✓) in the appropriate box.

**1. What is the primary function of the respiratory system?**

☐ A. To eliminate oxygen from body cells.

☐ B. To supply body cells with carbon dioxide.

☐ C. To deliver oxygen to the blood and eliminate carbon dioxide.

☐ D. To deliver carbon dioxide to the blood and eliminate oxygen.

**2. Gas exchange occurs primarily in the:**

☐ A. Nose

☐ B. Alveoli

☐ C. Trachea

☐ D. Bronchi

**3. The normal respiratory rate for a healthy adult is:**

☐ A. 5–10 breaths/minute

☐ B. 12–20 breaths/minute

☐ C. 20–30 breaths/minute

☐ D. 40–50 breaths/minute

**4. The respiratory system consists of:**

☐ A. Lungs and trachea

☐ B. Lungs, trachea, and bronchi

☐ C. Larynx and bronchi

☐ D. Larynx, trachea, bronchi, and lungs

**5. The right lung consists of:**

☐ A. One lobe

☐ B. Two lobes

☐ C. Three lobes

☐ D. Four lobes

**6. The left lung consists of:**

☐ A. One lobe

☐ B. Two lobes

☐ C. Three lobes

☐ D. Four lobes

**7. The normal arterial oxygen saturation (SpO₂) is:**

☐ A. 90–100%

☐ B. 60–80%

☐ C. 50–90%

☐ D. 60–90%

**8. During arterial blood gas (ABG) sampling, the radial artery is punctured with the needle inserted at approximately:**

☐ A. 90°

☐ B. 15°

☐ C. 45°

☐ D. 60°

**9. The normal arterial blood pH is:**

☐ A. 7.35–7.45

☐ B. 7.25–7.55

☐ C. 6.35–6.45

☐ D. 7.15–7.25

**10. The normal arterial partial pressure of carbon dioxide (PaCO₂) is:**

☐ A. 35–45 mmHg

☐ B. 35–55 mmHg

☐ C. 45–65 mmHg

☐ D. 35–41 mmHg

**11. The normal serum bicarbonate (HCO₃⁻) level is:**

☐ A. 21–25 mEq/L

☐ B. 22–35 mEq/L

☐ C. 21–35 mEq/L

☐ D. 21–22 mEq/L

**12. The normal arterial partial pressure of oxygen (PaO₂) is:**

☐ A. 80–100 mmHg

☐ B. 70–100 mmHg

☐ C. 60–90 mmHg

☐ D. 50–90 mmHg

**Section II. Mechanical Ventilation (19 Items)**

**1. Which of the following is an absolute indication for mechanical ventilation?**

☐ A. Injury to the respiratory center in the brain

☐ B. After all types of surgery

☐ C. After inguinal hernia surgery

☐ D. After tonsillectomy

**2. Which of the following is an elective indication for mechanical ventilation?**

☐ A. Respiratory muscle injury

☐ B. Injury to the respiratory center in the brain

☐ C. Respiratory diseases

☐ D. All of the above

**3. Indications for mechanical ventilation include all of the following except:**

☐ A. Respiratory arrest

☐ B. Coma and post-cardiopulmonary resuscitation

☐ C. Otitis media

☐ D. Spinal cord injury and head trauma

**4. Mechanical ventilation is defined as:**

☐ A. Replacing or supporting normal lung function using a mechanical ventilator

☐ B. A life-support system used to replace or support cardiac function

☐ C. Replacing spontaneous breathing by inserting an endotracheal tube only

☐ D. Supporting lung function for a short period only

**5. The goals of mechanical ventilation include all of the following except:**

☐ A. Improving blood oxygenation

☐ B. Improving blood pressure

☐ C. Reducing intracranial pressure by controlling ventilation

☐ D. Preventing atelectasis

**6. Which of the following is an indication for initiating mechanical ventilation?**

☐ A. Rapid, shallow breathing (>35 breaths/min)

☐ B. PaO₂ less than 60 mmHg

☐ C. Cyanosis, profuse sweating, and altered level of consciousness

☐ D. All of the above

**7. Endotracheal suctioning should be performed:**

☐ A. Only when clinically indicated

☐ B. Whenever the ventilator alarm sounds

☐ C. Whenever the patient has retained secretions

☐ D. Continuously using a closed suction system

**8. Hyperoxygenation during endotracheal suctioning should be provided:**

☐ A. Before and after suctioning

☐ B. During and after suctioning

☐ C. Only after suctioning

☐ D. Only before suctioning

**9. All of the following are components of endotracheal tube care except:**

☐ A. Ensuring correct tube position

☐ B. Assessing the mouth for pressure ulcers and providing oral care

☐ C. Avoiding suctioning when indicated

☐ D. Monitoring cuff pressure

**10. To prevent ventilator-associated pneumonia (VAP), the nurse should:**

☐ A. Elevate the head of the bed to **30–45°**.

☐ B. Perform regular oral care with **chlorhexidine**.

☐ C. Assess the patient's readiness for extubation daily.

☐ D. **All of the above.**

**11. Which of the following is a type of mechanical ventilation?**

☐ A. Non-invasive mechanical ventilation (NIMV)

☐ B. T-piece

☐ C. Continuous Positive Airway Pressure (CPAP)

☐ D. All of the above

**12. Which of the following are ventilator modes?**

☐ A. Synchronized Intermittent Mandatory Ventilation (SIMV)

☐ B. Assist-Control Ventilation (ACV)

☐ C. Continuous Mandatory Ventilation (CMV)

☐ D. All of the above

**13. Which ventilator mode allows spontaneous breathing between mandatory breaths?**

☐ A. SIMV

☐ B. ACV

☐ C. CPAP

☐ D. CMV

**14. A high airway pressure alarm may occur because of:**

☐ A. The patient biting the endotracheal tube

☐ B. Kinking of the endotracheal tube or ventilator tubing

☐ C. Airway secretions requiring suctioning

☐ D. Patient–ventilator asynchrony

☐ E. All of the above

**15. A low airway pressure alarm may occur because of:**

☐ A. Ventilator circuit disconnection

☐ B. Leakage in the humidifier or tubing

☐ C. Inadequately inflated endotracheal tube cuff

☐ D. All of the above

**16. Which of the following is NOT a complication of mechanical ventilation?**

☐ A. Ventilator-associated pneumonia

☐ B. Pneumothorax

☐ C. Lung abscess

☐ D. Renal colic

**17. Oxygen toxicity is most likely to occur when:**

☐ A. FiO₂ exceeds 60% for more than 48 hours

☐ B. FiO₂ is less than 60% for more than 48 hours

☐ C. FiO₂ exceeds 60% for more than 12 hours

☐ D. FiO₂ exceeds 60% for more than 6 hours

**18. Before initiating mechanical ventilation, the nurse should:**

☐ A. Prepare and check the ventilator

☐ B. Connect the ventilator to the oxygen and power sources

☐ C. Prepare the airway and suction equipment

☐ D. All of the above

**19. During and after initiation of mechanical ventilation, the nurse should:**

☐ A. Monitor vital signs and ventilator parameters

☐ B. Assess the patient's color and humidifier function

☐ C. Perform suctioning as needed and provide routine nursing care

☐ D. All of the above

**Section III. Mechanical Ventilator, Modes, Alarms, Settings, Complications, and Nursing Care**

**Mechanical Ventilator Settings**

**1. Pressure Support Ventilation (PSV) is primarily used to:**

☐ A. Increase peak inspiratory pressure (PIP)

☐ B. Reduce oxygen requirements

☐ C. Decrease the work of breathing

☐ D. Prevent alveolar collapse

**2. In pressure-cycled ventilation, which parameter is predetermined?**

☐ A. Pressure

☐ B. Time

☐ C. Tidal volume

☐ D. Flow rate

**3. The recommended tidal volume for mechanically ventilated adult patients is:**

☐ A. 5–7 mL/kg

☐ B. 7–9 mL/kg

☐ C. 5–12 mL/kg

☐ D. 10–15 mL/kg

**Complications of Mechanical Ventilation**

**4. Positive-pressure mechanical ventilation affects the cardiovascular system because it:**

☐ A. Increases cardiac output

☐ B. Decreases venous return to the heart

☐ C. Increases blood pressure

☐ D. Increases venous return to the heart

**5. Which of the following is a sign of pulmonary barotrauma caused by mechanical ventilation?**

☐ A. Sudden lethargy

☐ B. Increased breath sounds over the affected lung

☐ C. Increased blood pressure

☐ D. Increased coughing associated with a high airway pressure alarm

**6. Positive-pressure ventilation may increase intracranial pressure (ICP) by:**

☐ A. Decreasing intrathoracic pressure

☐ B. Increasing cerebral perfusion

☐ C. Reducing venous drainage from the brain

☐ D. Increasing mean arterial pressure

**7. Which of the following renal effects may occur during mechanical ventilation?**

☐ A. No effect on urine output

☐ B. Decreased urine output

☐ C. Increased urine output

☐ D. Unknown effect

**Nursing Care of Patients Receiving Mechanical Ventilation**

**8. In an emergency, the fastest method to establish an airway is:**

☐ A. Oral endotracheal intubation

☐ B. Nasotracheal intubation

☐ C. Tracheostomy

☐ D. Oropharyngeal airway

**9. Which of the following complications may occur in patients with an endotracheal tube?**

☐ A. Infection

☐ B. Aspiration pneumonia

☐ C. Gastric ulcer

☐ D. A and B only

**10. During endotracheal tube removal (extubation), the patient may experience:**

☐ A. Indigestion

☐ B. Aspiration

☐ C. Vomiting

☐ D. B and C only

**11. To verify the correct position of the endotracheal tube, the nurse should:**

☐ A. Auscultate breath sounds

☐ B. Obtain a chest X-ray

☐ C. Check the centimeter marking at the lips

☐ D. All of the above

**12. Nursing care for a patient with an endotracheal tube should include:**

☐ A. Deflating the cuff every 8 hours

☐ B. Suctioning the oral cavity as needed

☐ C. Repositioning the patient every hour

☐ D. Measuring vital signs only

**13. Which of the following indicates accidental displacement of the endotracheal tube?**

☐ A. Vomiting

☐ B. Loss of appetite

☐ C. Audible vocal sounds

☐ D. Nasal secretions

**14. The oral airway should be changed every:**

☐ A. 2–4 hours

☐ B. 4–8 hours

☐ C. 8–12 hours

☐ D. 24–48 hours

**15. The normal saline solution used to rinse the suction catheter should be changed:**

☐ A. Every 2–4 hours

☐ B. Every 48 hours

☐ C. Every 8–24 hours

☐ D. After each suctioning procedure

**16. The maximum duration of endotracheal suctioning should be:**

☐ A. 15 seconds

☐ B. 20 seconds

☐ C. 30 seconds

☐ D. 60 seconds

**17. To prevent hypoxemia during endotracheal suctioning, the nurse should:**

☐ A. Position the patient in a sitting position

☐ B. Record vital signs only

☐ C. Hyperoxygenate the patient before suctioning

☐ D. Encourage deep-breathing exercises

**18. When a low exhaled tidal volume alarm sounds, the nurse should first:**

☐ A. Notify the physician

☐ B. Check the ventilator circuit connections

☐ C. Provide manual ventilation

☐ D. Inflate the endotracheal tube cuff

**19. When a low airway pressure alarm sounds, the nurse should first:**

☐ A. Increase the alarm volume and suction the patient

☐ B. Check for leaks in the ventilator tubing

☐ C. Assess the endotracheal tube cuff

☐ D. Verify that the patient remains connected to the ventilator

**20. Ventilator tubing, humidifiers, and breathing circuits should be routinely changed every:**

☐ A. 12 hours

☐ B. 24 hours

☐ C. 48 hours

☐ D. 72 hours

**Part IV: Nurses' Knowledge Regarding Weaning from Mechanical Ventilation**

**1. Weaning from mechanical ventilation is defined as:**

☐ A. Gradual withdrawal of ventilatory support provided by the mechanical ventilator.

☐ B. Sudden withdrawal of ventilatory support provided by the mechanical ventilator.

☐ C. Removal of the endotracheal tube.

☐ D. Removal of the oxygen mask

**2. Readiness for weaning from mechanical ventilation includes:**

☐ A. Presence of respiratory tract infection.

☐ B. Hemodynamic stability with acceptable arterial blood gas values.

☐ C. Bronchospasm.

☐ D. Hemodynamic instability.

**3. Which of the following is considered a criterion for initiating weaning?**

☐ A. The patient is awake, alert, and cooperative.

☐ B. The patient is able to initiate spontaneous breathing with minimal effort.

☐ C. The patient has no fever, severe pain, cyanosis, or cardiac arrhythmias and has adequate nutritional status.

☐ D. All of the above.

**4. Which of the following are methods of weaning from mechanical ventilation?**

☐ A. T-piece trial.

☐ B. Synchronized Intermittent Mandatory Ventilation (SIMV).

☐ C. Continuous Positive Airway Pressure (CPAP).

☐ D. All of the above.

**5. The recommended duration of a spontaneous breathing trial (SBT) is:**

☐ A. Less than 30 minutes.

☐ B. More than 120 minutes.

☐ C. More than 180 minutes.

☐ D. 30–120 minutes.

**6. Which of the following is a sign of weaning failure?**

☐ A. Tachypnea and respiratory distress.

☐ B. Cardiac arrhythmia.

☐ C. Use of accessory respiratory muscles.

☐ D. All of the above.

**7. A possible complication after unsuccessful weaning is:**

☐ A. Reconnection to mechanical ventilation.

☐ B. Hypertension.

☐ C. Hypotension.

☐ D. Regular spontaneous breathing.

**8. Factors that may interfere with successful weaning include:**

☐ A. Psychological factors.

☐ B. Abdominal distension.

☐ C. Poor nutrition and inadequate rest.

☐ D. All of the above.

**9. Factors that improve successful weaning include:**

☐ A. Good psychological status.

☐ B. Adequate nutrition.

☐ C. Adequate oxygen saturation.

☐ D. All of the above.

**10. Positive End-Expiratory Pressure (PEEP) is used to:**

☐ A. Prevent alveolar collapse at the end of expiration.

☐ B. Empty the lungs during expiration.

☐ C. Provide continuous pressure during both inspiration and expiration.

☐ D. Prevent alveolar collapse at the end of inspiration.

**11. Continuous Positive Airway Pressure (CPAP) is used when:**

☐ A. The patient is unable to breathe spontaneously.

☐ B. The patient is breathing spontaneously but requires continuous positive airway pressure.

☐ C. To prevent alveolar collapse at the end of expiration.

☐ D. To empty the lungs during expiration.

**12. An FiO₂ of 1.0 means that the patient is receiving:**

☐ A. 50% oxygen.

☐ B. 60% oxygen.

☐ C. 80% oxygen.

☐ D. 100% oxygen.

**13. Tidal Volume (VT) refers to:**

☐ A. The volume of air required to expand the lungs (6–10 mL/kg).

☐ B. The volume of air required to expand the lungs (9–20 mL/kg).

☐ C. The volume of air required to expand the lungs (12–24 mL/kg).

☐ D. The volume of air required to expand the lungs (12–30 mL/kg).

**14. Before initiating weaning, the tidal volume (VT) should be:**

☐ A. Greater than 8 mL/kg.

☐ B. Greater than 4 mL/kg.

☐ C. Greater than 5 mL/kg.

☐ D. Greater than 3 mL/kg.

**15. Before initiating weaning, the Negative Inspiratory Pressure (NIP) should be:**

☐ A. Greater than −20 cmH₂O.

☐ B. Greater than −30 cmH₂O.

☐ C. Greater than −40 cmH₂O.

☐ D. Greater than −50 cmH₂O.

**16. Before initiating weaning, the fraction of inspired oxygen (FiO₂) should generally be:**

☐ A. **≤ 40%**

☐ B. ≤ 60%

☐ C. ≤ 80%

☐ D. 100%

**17. Before initiating weaning, the respiratory rate (RR) should be:**

☐ A. Less than 30 breaths/minute.

☐ B. Less than 40 breaths/minute.

☐ C. Less than 50 breaths/minute.

☐ D. Less than 60 breaths/minute.

**18. Before initiating weaning, Positive End-Expiratory Pressure (PEEP) should be:**

☐ A. Less than 9 cmH₂O.

☐ B. Less than 10 cmH₂O.

☐ C. Less than or equal to 8 cmH₂O.

☐ D. Less than 5 cmH₂O.

**19. During weaning, the patient's blood pressure should not increase by more than:**

☐ A. 20% above baseline.

☐ B. 30% above baseline.

☐ C. 40% above baseline.

☐ D. 50% above baseline.

**20. During weaning, the patient's heart rate should not increase by more than:**

☐ A. 20% above baseline.

☐ B. 30% above baseline.

☐ C. 40% above baseline.

☐ D. 50% above baseline.

**21. Infection control measures during weaning include:**

☐ A. Hand hygiene before and after patient care.

☐ B. Wearing gloves, gown, and mask.

☐ C. Using aseptic technique during procedures.

☐ D. All of the above.

**22. Nursing care during weaning from mechanical ventilation includes:**

☐ A. Continuous monitoring of vital signs.

☐ B. Monitoring arterial blood gases.

☐ C. Assessing respiratory effort, skin color, and level of consciousness.

☐ D. All of the above.

**Answer Key**

**Section I. Anatomy and Physiology of the Respiratory System (12 items)**

| **Item** | **Correct Answer** |
| --- | --- |
| 1 | C |
| 2 | B |
| 3 | B |
| 4 | D |
| 5 | C |
| 6 | B |
| 7 | A |
| 8 | B |
| 9 | A |
| 10 | A |
| 11 | A |
| 12 | A |

**Section II. Mechanical Ventilation (19 items)**

| **Item** | **Correct Answer** |
| --- | --- |
| 1 | A |
| 2 | D |
| 3 | C |
| 4 | A |
| 5 | B |
| 6 | D |
| 7 | A |
| 8 | A |
| 9 | C |
| 10 | D |
| 11 | D |
| 12 | D |
| 13 | A |
| 14 | E |
| 15 | D |
| 16 | D |
| 17 | A |
| 18 | D |
| 19 | D |

**Section III. Mechanical Ventilator, Modes, Alarms, Settings, Complications, and Nursing Care (20 items)**

| **Item** | **Correct Answer** |
| --- | --- |
| 1 | C |
| 2 | A |
| 3 | A |
| 4 | B |
| 5 | D |
| 6 | C |
| 7 | B |
| 8 | A |
| 9 | D |
| 10 | D |
| 11 | D |
| 12 | B |
| 13 | C |
| 14 | D |
| 15 | D |
| 16 | A |
| 17 | C |
| 18 | B |
| 19 | D |
| 20 | D |

**Section IV. Weaning from Mechanical Ventilation (22 items)**

| **Item** | **Correct Answer** |
| --- | --- |
| 1 | A |
| 2 | B |
| 3 | D |
| 4 | D |
| 5 | D |
| 6 | D |
| 7 | A |
| 8 | D |
| 9 | D |
| 10 | A |
| 11 | B |
| 12 | D |
| 13 | A |
| 14 | C |
| 15 | A |
| 16 | A |
| 17 | A |
| 18 | C |
| 19 | A |
| 20 | A |
| 21 | D |
| 22 | D |

For each domain, the same scoring method was applied:

1. **Anatomy and Physiology of the Respiratory System (12 items)**
   - Score range: 0–12 points
   - Correct answer = 1 point; incorrect answer = 0 points.
2. **Mechanical Ventilation (19 items)**
   - Score range: 0–19 points
   - Correct answer = 1 point; incorrect answer = 0 points.
3. **Mechanical Ventilator Modes, Alarms, Settings, Complications, and Nursing Care (20 items)**
   - Score range: 0–20 points
   - Correct answer = 1 point; incorrect answer = 0 points.
4. **Weaning from Mechanical Ventilation (22 items)**
   - Score range: 0–22 points
   - Correct answer = 1 point; incorrect answer = 0 points.

The total knowledge score was calculated by summing the scores of all items, with a possible maximum score of **73 points**. The pre- and post-program knowledge scores were compared to evaluate the effectiveness of the educational intervention.
