# Supplementary material for: Enhancing nurses’ competence in mechanical ventilation: a quasi-experimental study and its association with patient severity using APACHE II
Source: Front Med (Lausanne). 2026 Aug 27;13:1891958. doi: 10.3389/fmed.2026.1891958 (PMC13587796; doi:10.3389/fmed.2026.1891958)
Supplement: Supplementary file 2 [file Data_Sheet_2.docx]

**Tool III: Nurses’ Practice Observation Checklist – Care of Patients on Mechanical Ventilators**

**Skill No. (1): Hand Washing**

| **No.** | **Steps** | **Done** | **Not Done** | **Comment** |
| --- | --- | --- | --- | --- |
| 1 | Remove jewelry, if possible, and secure it in a safe place. A plain wedding band may remain in place. | ☐ | ☐ |  |
| 2 | Check the product labeling for the correct amount of product needed. | ☐ | ☐ |  |
| 3 | Apply the correct amount of product to the palm of one hand. | ☐ | ☐ |  |
| 4 | Rub hands together, covering all surfaces of the hands, fingers, and between the fingers. | ☐ | ☐ |  |
| 5 | Clean the fingertips and the area beneath the fingernails. | ☐ | ☐ |  |
| 6 | Rub hands together until they are dry (at least **15 seconds**). | ☐ | ☐ |  |
| 7 | Apply oil-free lotion to the hands if desired. | ☐ | ☐ |  |

**Skill No (2): Suctioning of the endotracheal tube**

**Closed System**

| **No.** | **Comment** | **Done** | **Not**  **Done** | **Comment** |
| --- | --- | --- | --- | --- |
| **Assessment** |  |  |  |  |
| 1 | Assess lung sounds. | ☐ | ☐ |  |
| 2 | Assess oxygen saturation level. | ☐ | ☐ |  |
| 3 | Assess respiratory status. | ☐ | ☐ |  |
| 4 | Assess the patient for signs of respiratory distress such as nasal flaring, retractions, distress, or grunting. | ☐ | ☐ |  |
| **Implementation** |  |  |  |  |
| 5 | Bring necessary equipment to the bedside stand or over-bed table. | ☐ | ☐ |  |
| 6 | Perform hand hygiene. | ☐ | ☐ |  |
| 7 | Put on PPE, if indicated. | ☐ | ☐ |  |
| 8 | Identify the patient. | ☐ | ☐ |  |
| 9 | Close curtains around the bed and close the room door, if possible. | ☐ | ☐ |  |
| 10 | Determine the need for suctioning. Verify the suction order in the patient's chart. | ☐ | ☐ |  |
| 11 | Assess for pain or the potential to cause pain. | ☐ | ☐ |  |
| 12 | Administer pain medication, as prescribed, before suctioning. | ☐ | ☐ |  |
| 13 | Explain the procedure to the patient, even if the patient does not appear to be alert. | ☐ | ☐ |  |
| 14 | Reassure the patient that you will interrupt the procedure if he or she indicates respiratory difficulty. | ☐ | ☐ |  |
| 15 | Adjust bed to a comfortable working position. | ☐ | ☐ |  |
| 16 | Lower side rail closest to you. | ☐ | ☐ |  |
| 17 | Position the patient appropriately (Semi-Fowler's if conscious; lateral if unconscious). | ☐ | ☐ |  |
| 18 | Move the over-bed table close to your work area. | ☐ | ☐ |  |
| 19 | Turn suction to **100–120 mmHg** for an adult. | ☐ | ☐ |  |
| 20 | Open the package of the closed suction device using aseptic technique. | ☐ | ☐ |  |
| 21 | Put on sterile gloves. | ☐ | ☐ |  |
| 22 | Disconnect the ventilator from the endotracheal tube while maintaining sterility. | ☐ | ☐ |  |
| 23 | Connect the closed suction device to the endotracheal tube. | ☐ | ☐ |  |
| 24 | Reconnect the ventilator tubing while maintaining sterility. | ☐ | ☐ |  |
| 25 | Attach suction tubing to the suction catheter. | ☐ | ☐ |  |
| 26 | Prepare the sterile normal saline dosette. | ☐ | ☐ |  |
| 27 | Hyperventilate the patient using the ventilator sigh button before suctioning. | ☐ | ☐ |  |
| 28 | Turn the safety cap on the suction button. | ☐ | ☐ |  |
| 29 | Insert the suction catheter gently into the endotracheal tube. | ☐ | ☐ |  |
| 30 | Advance the catheter to the predetermined length. | ☐ | ☐ |  |
| 31 | Apply intermittent suction. | ☐ | ☐ |  |
| 32 | Rotate the catheter while withdrawing it (10–15 seconds maximum). | ☐ | ☐ |  |
| 33 | Hyperoxygenate or hyperventilate the patient, as ordered. | ☐ | ☐ |  |
| 34 | Flush the catheter with sterile normal saline until clean. | ☐ | ☐ |  |
| 35 | Wait 30 seconds to 1 minute before repeating suctioning if needed (maximum three passes). | ☐ | ☐ |  |
| 36 | Withdraw the catheter completely into the sheath and engage the safety button. | ☐ | ☐ |  |
| 37 | Remove the saline dosette and recap the irrigation port. | ☐ | ☐ |  |
| 38 | Suction the oral cavity with a separate catheter and perform oral hygiene. | ☐ | ☐ |  |
| 39 | Remove gloves and turn off suction. | ☐ | ☐ |  |
| 40 | Assist the patient to a comfortable position. | ☐ | ☐ |  |
| 41 | Raise the bed rail and place the bed in the lowest position. | ☐ | ☐ |  |
| 42 | Reassess the patient's respiratory status. | ☐ | ☐ |  |
| 43 | Remove PPE (if used) and perform hand hygiene. | ☐ | ☐ |  |
| **Documentation** |  |  |  |  |
| 44 | Document: time of suctioning, pre- and post-assessment findings, reason for suctioning, oxygen saturation level, and the characteristics and amount of secretions. | ☐ | ☐ |  |

**Skill No. (3): Nursing Care of the Endotracheal Tube**

| **No.** | **Steps** | **Done** | **Not Done** | **Comment** |
| --- | --- | --- | --- | --- |
| **Assessment** |  |  |  |  |
| 1 | Assess the need for retaping. | ☐ | ☐ |  |
| 2 | Assess the endotracheal tube length. | ☐ | ☐ |  |
| 3 | Assess lung sounds to obtain a baseline. | ☐ | ☐ |  |
| 4 | Assess oxygen saturation level. | ☐ | ☐ |  |
| 5 | Assess the chest for symmetric rise and fall during respiration. | ☐ | ☐ |  |
| **Implementation** |  |  |  |  |
| 6 | Bring necessary equipment to the bedside stand or over-bed table. | ☐ | ☐ |  |
| 7 | Perform hand hygiene and put on PPE, if indicated. | ☐ | ☐ |  |
| 8 | Identify the patient. | ☐ | ☐ |  |
| 9 | Close curtains around the bed and close the room door, if possible. | ☐ | ☐ |  |
| 10 | Assess the need for endotracheal tube retaping. | ☐ | ☐ |  |
| 11 | Administer prescribed pain medication or sedation before retaping and explain the procedure to the patient. | ☐ | ☐ |  |
| 12 | Obtain assistance from a second person to stabilize the endotracheal tube. | ☐ | ☐ |  |
| 13 | Adjust the bed to a comfortable working height. | ☐ | ☐ |  |
| 14 | Lower the side rail closest to you. | ☐ | ☐ |  |
| 15 | Position the patient appropriately (Semi-Fowler's if conscious; lateral if unconscious). | ☐ | ☐ |  |
| 16 | Move the over-bed table close to your work area. | ☐ | ☐ |  |
| 17 | Place a trash receptacle within easy reach. | ☐ | ☐ |  |
| 18 | Put on a face shield (or goggles) and mask. | ☐ | ☐ |  |
| 19 | Suction the patient. | ☐ | ☐ |  |
| 20 | Measure and prepare the first piece of tape. | ☐ | ☐ |  |
| 21 | Prepare the second piece of tape. | ☐ | ☐ |  |
| 22 | Wrap tape around two 3-mL syringes or tongue blades. | ☐ | ☐ |  |
| 23 | Pass one syringe/tongue blade behind the patient's neck. | ☐ | ☐ |  |
| 24 | Put on disposable gloves and have the assistant wear gloves. | ☐ | ☐ |  |
| 25 | Provide oral care, including suctioning the oral cavity. | ☐ | ☐ |  |
| 26 | Note the tube depth markings and begin removing the old tape while the assistant stabilizes the tube. | ☐ | ☐ |  |
| 27 | Remove the remaining tape and move the tube to the opposite side of the mouth (if orally intubated). | ☐ | ☐ |  |
| 28 | Assess the mouth for skin breakdown. | ☐ | ☐ |  |
| 29 | Verify that the endotracheal tube markings remain at the correct depth. | ☐ | ☐ |  |
| 30 | Remove old adhesive and clean the patient's face and neck. | ☐ | ☐ |  |
| 31 | Apply a skin barrier to the face. | ☐ | ☐ |  |
| 32 | Apply the first side of the new tape and secure it to the cheek. | ☐ | ☐ |  |
| 33 | Secure the upper and lower tape pieces around the tube. | ☐ | ☐ |  |
| 34 | Complete taping on the opposite side and ensure the tube is secure. | ☐ | ☐ |  |
| 35 | Auscultate lung sounds. | ☐ | ☐ |  |
| 36 | Assess cyanosis, oxygen saturation, chest symmetry, tube stability, and tube depth. | ☐ | ☐ |  |
| 37 | If cuffed, check cuff pressure using a handheld pressure gauge. | ☐ | ☐ |  |
| 38 | Assist the patient to a comfortable position, raise the side rail, lower the bed, remove PPE, and perform hand hygiene. | ☐ | ☐ |  |
| **Documentation** |  |  |  |  |
| 39 | Document the depth of the endotracheal tube from the teeth or lips. | ☐ | ☐ |  |
| 40 | Document suctioned secretions (amount, consistency, color), skin and mucous membrane condition, lung sounds, oxygen saturation, skin color, cuff pressure, and chest symmetry. | ☐ | ☐ |  |

## **Skill No. (4): Providing Care to Patients During Weaning from Mechanical Ventilation**

| **No.** | **Steps** | **Done** | **Not Done** | **Comment** |
| --- | --- | --- | --- | --- |
| **Assessment** |  |  |  |  |
| 1 | Assess the patient's level of consciousness. | ☐ | ☐ |  |
| 2 | Assess the patient's nutritional status. | ☐ | ☐ |  |
| 3 | Assess the patient's comfort level. | ☐ | ☐ |  |
| 4 | Assess the patient's sleep pattern. | ☐ | ☐ |  |
| 5 | Assess for thoracic pain. | ☐ | ☐ |  |
| 6 | Assess metabolic status. | ☐ | ☐ |  |
| 7 | Assess body temperature (fever). | ☐ | ☐ |  |
| 8 | Assess for signs of infection. | ☐ | ☐ |  |
| 9 | Assess the amount of airway secretions. | ☐ | ☐ |  |
| 10 | Assess bowel function. | ☐ | ☐ |  |
| 11 | Assess hemodynamic stability. | ☐ | ☐ |  |
| 12 | Review laboratory investigation results. | ☐ | ☐ |  |
| 13 | Assess the patient's activity level. | ☐ | ☐ |  |
| 14 | Assess emotional status and anxiety level. | ☐ | ☐ |  |
| **Pulmonary Function Assessment** | |  |  |  |
| 15 | Assess minute ventilation (MV). | ☐ | ☐ |  |
| 16 | Assess maximal inspiratory pressure (MIP/NIF). | ☐ | ☐ |  |
| 17 | Assess maximal expiratory pressure (MEP). | ☐ | ☐ |  |
| 18 | Assess tidal volume (VT). | ☐ | ☐ |  |
| 19 | Assess vital capacity (VC). | ☐ | ☐ |  |
| 20 | Assess positive end-expiratory pressure (PEEP). | ☐ | ☐ |  |
| **Arterial Blood Gas Assessment** | |  |  |  |
| 21 | Assess arterial pH. | ☐ | ☐ |  |
| 22 | Assess PaO₂. | ☐ | ☐ |  |
| 23 | Assess PaCO₂. | ☐ | ☐ |  |
| 24 | Assess FiO₂ requirement. | ☐ | ☐ |  |
| 25 | Assess fluid and electrolyte balance. | ☐ | ☐ |  |
| **Preparation** |  |  |  |  |
| 26 | Explain the procedure and its purpose to the patient and family. | ☐ | ☐ |  |
| 27 | Encourage the patient to relax. | ☐ | ☐ |  |
| 28 | Ensure the patient understands the procedure. | ☐ | ☐ |  |
| 29 | Position the patient in an upright or semi-Fowler's position. | ☐ | ☐ |  |
| 30 | Prepare all necessary equipment. | ☐ | ☐ |  |
| 31 | Suction the airway if indicated. | ☐ | ☐ |  |
| 32 | Perform hand hygiene. | ☐ | ☐ |  |
| 33 | Don gloves and other PPE if indicated. | ☐ | ☐ |  |
| 34 | Verify the physician's order for the selected weaning method. | ☐ | ☐ |  |

## **Weaning Methods**

**A. T-Piece Weaning Trial**

| **No.** | **Steps** | **Done** | **Not Done** | **Comment** |
| --- | --- | --- | --- | --- |
| 35 | Remove the patient from the mechanical ventilator. | ☐ | ☐ |  |
| 36 | Connect the patient to a T-piece with humidified oxygen. | ☐ | ☐ |  |
| 37 | Instruct the patient to breathe normally. | ☐ | ☐ |  |
| 38 | Monitor respiratory rate. | ☐ | ☐ |  |
| 39 | Monitor heart rate. | ☐ | ☐ |  |
| 40 | Monitor blood pressure. | ☐ | ☐ |  |
| 41 | Monitor oxygen saturation (SpO₂). | ☐ | ☐ |  |
| 42 | Monitor ECG. | ☐ | ☐ |  |
| 43 | Observe the patient's general appearance. | ☐ | ☐ |  |
| 44 | Obtain arterial blood gases after 30 minutes, if indicated. | ☐ | ☐ |  |
| 45 | Suction the airway as needed. | ☐ | ☐ |  |
| 46 | Return the patient to full ventilatory support if signs of intolerance occur. | ☐ | ☐ |  |
| 47 | Assess for decreased level of consciousness. | ☐ | ☐ |  |
| 48 | Assess for SpO₂ < 90%. | ☐ | ☐ |  |
| 49 | Assess for use of accessory respiratory muscles. | ☐ | ☐ |  |
| 50 | Assess for dyspnea. | ☐ | ☐ |  |
| 51 | Assess for fatigue or chest pain. | ☐ | ☐ |  |
| 52 | Assess for diaphoresis. | ☐ | ☐ |  |
| 53 | Assess for anxiety. | ☐ | ☐ |  |
| 54 | Assess tidal volume < 5 mL/kg. | ☐ | ☐ |  |
| 55 | Assess respiratory rate > 30 breaths/min. | ☐ | ☐ |  |
| 56 | Assess heart rate increase > 20 beats/min from baseline. | ☐ | ☐ |  |
| 57 | Assess systolic blood pressure change ±20 mmHg or diastolic blood pressure >100 mmHg. | ☐ | ☐ |  |
| 58 | Notify the physician and prepare for extubation if the patient successfully completes the T-piece trial. | ☐ | ☐ |  |

**B. IMV/SIMV Weaning Method**

| **No.** | **Steps** | **Done** | **Not Done** | **Comment** |
| --- | --- | --- | --- | --- |
| 59 | Set the ventilator to SIMV mode. | ☐ | ☐ |  |
| 60 | Gradually decrease the mandatory respiratory rate according to protocol or physician's order. | ☐ | ☐ |  |
| 61 | Monitor the patient's response throughout the trial. | ☐ | ☐ |  |
| 62 | Obtain arterial blood gases after 30 minutes, if indicated. | ☐ | ☐ |  |
| 63 | Return the patient to full ventilatory support if intolerance occurs. | ☐ | ☐ |  |
| 64 | Notify the physician and prepare for extubation if weaning criteria are achieved. | ☐ | ☐ |  |

**C. Pressure Support Ventilation (PSV)**

| **No.** | **Steps** | **Done** | **Not Done** | **Comment** |
| --- | --- | --- | --- | --- |
| 65 | Initiate pressure support ventilation at the prescribed level. | ☐ | ☐ |  |
| 66 | Gradually reduce the pressure support level according to protocol or physician's order. | ☐ | ☐ |  |
| 67 | Monitor the patient's response throughout the trial. | ☐ | ☐ |  |
| 68 | Obtain arterial blood gases after 30 minutes, if indicated. | ☐ | ☐ |  |
| 69 | Return the patient to full ventilatory support if intolerance occurs. | ☐ | ☐ |  |
| 70 | Notify the physician when the target pressure support level is achieved. | ☐ | ☐ |  |

**D. CPAP Trial**

| **No.** | **Steps** | **Done** | **Not Done** | **Comment** |
| --- | --- | --- | --- | --- |
| 71 | Explain the purpose of CPAP therapy to the patient. | ☐ | ☐ |  |
| 72 | Switch the ventilator from resting settings to CPAP. | ☐ | ☐ |  |
| 73 | Instruct the patient to breathe normally. | ☐ | ☐ |  |
| 74 | Monitor the patient's response throughout the trial. | ☐ | ☐ |  |
| 75 | Obtain arterial blood gases after 30 minutes, if indicated. | ☐ | ☐ |  |
| 76 | Return the patient to full ventilatory support if intolerance occurs. | ☐ | ☐ |  |
| 77 | Notify the physician if the patient successfully completes the CPAP trial and prepare for extubation. | ☐ | ☐ |  |

**Documentation**

| **No.** | **Steps** | **Done** | **Not Done** | **Comment** |
| --- | --- | --- | --- | --- |
| 78 | Document the weaning mode or method used. | ☐ | ☐ |  |
| 79 | Document the duration of the weaning trial. | ☐ | ☐ |  |
| 80 | Document the level of ventilatory support (PSV, SIMV, or CPAP). | ☐ | ☐ |  |
| 81 | Document the patient's vital signs during each weaning interval. | ☐ | ☐ |  |
| 82 | Document arterial blood gas (ABG) results. | ☐ | ☐ |  |
| 83 | Document pulse oximetry (SpO₂) readings. | ☐ | ☐ |  |
| 84 | Document any unexpected outcomes or complications. | ☐ | ☐ |  |

**Post-Weaning Evaluation**

| **No.** | **Steps** | **Done** | **Not Done** | **Comment** |
| --- | --- | --- | --- | --- |
| 85 | Reassess the patient's level of consciousness. | ☐ | ☐ |  |
| 86 | Reassess respiratory rate and breathing pattern. | ☐ | ☐ |  |
| 87 | Reassess heart rate and blood pressure. | ☐ | ☐ |  |
| 88 | Reassess oxygen saturation (SpO₂). | ☐ | ☐ |  |
| 89 | Auscultate lung sounds. | ☐ | ☐ |  |
| 90 | Assess patient comfort and tolerance of weaning. | ☐ | ☐ |  |
| 91 | Assess the need for airway suctioning. | ☐ | ☐ |  |
| 92 | Assess readiness for extubation according to institutional protocol. | ☐ | ☐ |  |
| 93 | Maintain airway patency throughout the weaning process. | ☐ | ☐ |  |
| 94 | Provide reassurance and emotional support to the patient. | ☐ | ☐ |  |
| 95 | Communicate the patient's status to the physician and healthcare team. | ☐ | ☐ |  |
| 96 | Continue monitoring until the patient is stable or the physician's orders change. | ☐ | ☐ |  |
| 97 | Complete all nursing documentation and sign the clinical record. | ☐ | ☐ |  |

## **Skill No. (5): Arterial Blood Gas (ABG) Sampling**

| **No.** | **Steps** | **Done** | **Not Done** | **Comment** |
| --- | --- | --- | --- | --- |
| **Assessment** |  |  |  |  |
| 1 | Review the patient's medical record and plan of care for the need for an ABG specimen. | ☐ | ☐ |  |
| 2 | Assess heart rate. | ☐ | ☐ |  |
| 3 | Assess blood pressure. | ☐ | ☐ |  |
| 4 | Auscultate heart sounds. | ☐ | ☐ |  |
| 5 | Assess respiratory rate. | ☐ | ☐ |  |
| 6 | Assess chest excursion. | ☐ | ☐ |  |
| 7 | Auscultate lung sounds. | ☐ | ☐ |  |
| 8 | Assess oxygen therapy and oxygen flow rate, if prescribed. | ☐ | ☐ |  |
| 9 | Perform Allen's test to determine adequacy of peripheral circulation. | ☐ | ☐ |  |
| 10 | Assess the patient's understanding of the procedure. | ☐ | ☐ |  |
| 11 | Ask whether the patient has experienced fainting, sweating, or nausea during previous blood sampling. | ☐ | ☐ |  |
| **Implementation** |  |  |  |  |
| 12 | Gather the required equipment and check expiration dates. | ☐ | ☐ |  |
| 13 | Verify the physician's order for ABG analysis. | ☐ | ☐ |  |
| 14 | Confirm that the patient has not been suctioned within the previous 15 minutes. | ☐ | ☐ |  |
| 15 | Check the facility policy regarding local anesthesia. | ☐ | ☐ |  |
| 16 | Administer local anesthetic if prescribed. | ☐ | ☐ |  |
| 17 | Bring equipment to the bedside. | ☐ | ☐ |  |
| 18 | Perform hand hygiene. | ☐ | ☐ |  |
| 19 | Don appropriate PPE. | ☐ | ☐ |  |
| 20 | Verify the patient's identity using two identifiers. | ☐ | ☐ |  |
| 21 | Explain the procedure to the patient. | ☐ | ☐ |  |
| 22 | Ensure privacy. | ☐ | ☐ |  |
| 23 | Verify specimen labels. | ☐ | ☐ |  |
| 24 | Provide adequate lighting. | ☐ | ☐ |  |
| 25 | Place a waste container nearby. | ☐ | ☐ |  |
| 26 | Position the patient correctly. | ☐ | ☐ |  |
| 27 | Place a waterproof pad and rolled towel beneath the wrist. | ☐ | ☐ |  |
| 28 | Ask the patient to clench the fist during Allen's test. | ☐ | ☐ |  |
| 29 | Compress both the radial and ulnar arteries. | ☐ | ☐ |  |
| 30 | Ask the patient to relax and open the hand. | ☐ | ☐ |  |
| 31 | Release pressure from the ulnar artery and evaluate hand perfusion. | ☐ | ☐ |  |
| 32 | Put on gloves and palpate the radial artery. | ☐ | ☐ |  |
| 33 | Clean the puncture site using antiseptic solution. | ☐ | ☐ |  |
| 34 | Stabilize the wrist over the rolled towel. | ☐ | ☐ |  |
| 35 | Palpate the artery with the nondominant hand. | ☐ | ☐ |  |
| 36 | Insert the needle bevel-up at the correct angle. | ☐ | ☐ |  |
| 37 | Puncture the artery and allow the syringe to fill spontaneously. | ☐ | ☐ |  |
| 38 | Withdraw the needle and immediately apply pressure with sterile gauze. | ☐ | ☐ |  |
| 39 | Maintain pressure for the recommended time. | ☐ | ☐ |  |
| 40 | Apply an adhesive bandage or pressure dressing. | ☐ | ☐ |  |
| 41 | Remove any air bubbles from the specimen. | ☐ | ☐ |  |
| 42 | Activate the needle safety device and cap the syringe. | ☐ | ☐ |  |
| 43 | Gently roll the syringe to mix the blood with heparin. | ☐ | ☐ |  |
| 44 | Label the specimen according to facility policy. | ☐ | ☐ |  |
| 45 | Place the specimen in an iced biohazard transport bag. | ☐ | ☐ |  |
| 46 | Dispose of sharps safely. | ☐ | ☐ |  |
| 47 | Remove gloves and PPE. | ☐ | ☐ |  |
| 48 | Perform hand hygiene. | ☐ | ☐ |  |
| 49 | Transport the specimen to the laboratory immediately. | ☐ | ☐ |  |

### **Scoring System of the Observational Checklist**

| **Skill** | **Score Range** | **Scoring Method** |
| --- | --- | --- |
| Hand Washing | 0–7 | Each correctly performed step = **1**; incorrectly performed or omitted step = **0**. |
| Suctioning of the Endotracheal Tube | 0–44 | Each correctly performed step = **1**; incorrectly performed or omitted step = **0**. |
| Endotracheal Tube Care | 0–39 | Each correctly performed step = **1**; incorrectly performed or omitted step = **0**. |
| Providing Care to Patients During Weaning from Mechanical Ventilation | 0–97 | Each correctly performed step = **1**; incorrectly performed or omitted step = **0**. |
| Arterial Blood Gas (ABG) Sampling | 0–49 | Each correctly performed step = **1**; incorrectly performed or omitted step = **0**. |
| **Overall Practice** | **0–236** | Sum of the scores of all five skills. Higher scores indicate better nursing performance. |
